# Causal association between major depressive disorder and venous thromboembolism: a bidirectional mendelian randomization study

**DOI:** 10.3389/fgene.2024.1383333

**Published:** 2024-06-25

**Authors:** Hong-Yan Li, Li-Hong Wang, Jing Wang, Yong-Bo Wang, Hai-Shan Wang

**Affiliations:** ^1^ Department of Pharmacy, Qingdao University Medical College Affiliated Yantai Yuhuangding Hospital, Yantai, China; ^2^ Department of Ultrasound, Qingdao University Medical College Affiliated Yantai Yuhuangding Hospital, Yantai, China; ^3^ Center for Evidence-Based and Translational Medicine, Zhongnan Hospital of Wuhan University, Wuhan, China; ^4^ Department of Intensive Care Unit, Yantai Yeda Hospital, Yantai, China

**Keywords:** major depressive disorder, venous thromboembolism, risk factor, singlenucleotide polymorphisms, mendelian randomization

## Abstract

**Purpose:**

Major depressive disorder (MDD) and venous thromboembolism (VTE) may be linked in observational studies. However, the causal association remains ambiguous. Therefore, this study investigates the causal associations between them.

**Methods:**

We performed a two-sample univariable and multivariable bidirectional Mendelian randomization (MR) analysis to evaluate the associations between MDD and VTE. The summary genetic associations of MDD statistics were obtained from the Psychiatric Genomics Consortium and UK Biobank. Information on VTE, deep vein thrombosis (DVT), and pulmonary embolism (PE) were obtained from the FinnGen Biobank. Inverse-variance weighting was used as the main analysis method. Other methods include weighted median, MR-Egger, Simple mode, and Weighted mode.

**Results:**

Univariable MR analysis revealed no significant associations between MDD and VTE risk (odds ratio (OR): 0.936, 95% confidence interval (CI): 0.736–1.190, *p* = 0.590); however, after adjusting the potential relevant polymorphisms of body mass index and education, the multivariable MR analysis showed suggestive evidence of association between them (OR: 1.163, 95% CI: 1.004–1.346, *p* = 0.044). Univariable MR analysis also revealed significant associations between MDD and PE risk (OR: 1.310, 95% CI: 1.073–1.598, *p* = 0.008), but the association between them was no longer significant in MVMR analysis (*p* = 0.072). We found no significant causal effects between MDD and DVT risk in univariable or multivariable MR analyses. There was also no clear evidence showing the causal effects between VTE, PE, or DVT and MDD risk.

**Conclusion:**

We provide suggestive genetic evidence to support the causal association between MDD and VTE risk. No causal associations were observed between VTE, PE, or DVT and MDD risk. Further validation of these associations and investigations of potential mechanisms are required.

## 1 Introduction

Venous thromboembolism (VTE), including deep vein thrombosis (DVT) and pulmonary embolism (PE), is a multifactorial disorder disease with hereditary and acquired risk factors (Rosendaal, 1999). VTE has a high annual incidence of 1–2 persons per 1,000 individuals and is a major contributor to the global disease burden (Wendelboe and Raskob, 2016; Khan et al., 2021). Any cause that can lead to venous endothelial injury, stagnant venous blood flow, and a hypercoagulable state of the blood is a risk factor for VTE (Piazza, 2015). Regarding the risk factors for VTE, in addition to the identified risk factors such as trauma, surgery, obesity, and genetic susceptibility to thrombosis, more unidentified factors may be related to VTE etiology (Pastori et al., 2023). Identifying high-risk patients for early risk stratification of patients may result in more effective treatment strategies (Barco et al., 2016; Barco et al., 2020).

Major depressive disorder (MDD) is a psychological disorder that changes an individual’s emotional state, leading to diminished experience of positive emotions, cognitive impairments, and poor concentration; it is a serious threat to the physical and mental health of individuals (Dehn and Beblo, 2019). Increasing evidence shows that depression and prothrombotic states are associated with dysfunction of the stress response system (Gold, 2015; Hunter et al., 2017; Sandrini et al., 2020). Several common mechanistic pathways, such as increased platelet activation, procoagulant activity, endothelial dysfunction, and inflammatory processes, may trigger the development of depression and VTE (Gold, 2015). Despite the plausibility of behavioral and biological mechanisms, the existing findings are inconsistent. In a prospective study, the risk of venous thrombosis was 1.6 times higher in patients with depression than in those without depression (Austin et al., 2013). However, a large prospective study showed that VTE risk was not significantly increased in women who reported depression or anxiety (Parkin et al., 2017). Concurrently, there may be a reverse causality between depression and VTE; a previous study showed a 2.35-fold increased risk of depression in individuals with VTE compared with the general population [9]. To date, based on the inconsistent results derived from observational studies, the relationship between MDD and VTE remains unclear. Additionally, previous studies investigating the relationship between MDD and VTE were observational, and conventional observational studies have difficulty determining whether noted correlations are causal because of limitations, including potential confounders, reversal causality bias, and measurement error (Sekula et al., 2016). Therefore, further evidence is required to elucidate the causal relationship between depression and VTE and the pathways that are potentially involved.

Mendelian randomization (MR) is a statistical method based on genetic variation that uses genetic variation already present in nature as an instrumental variable (IV) to simulate randomly assigned experimental conditions to investigate the causal association between risk factors and disease development (Sekula et al., 2016; Emdin et al., 2017). The central idea is that genetic variation is randomly distributed among individuals and is independent of environmental and behavioral factors. By exploiting the association between these genetic variants and the exposure factor of interest, the causal effect of that exposure on a particular outcome can be inferred (Lawlor et al., 2008). Therefore, this study aimed to investigate the causal associations between MDD and VTE (including DVT and PE) using univariable and multivariable bidirectional two-sample MR analyses.

## 2 Materials and methods

### 2.1 Study design

**FIGURE 1:**
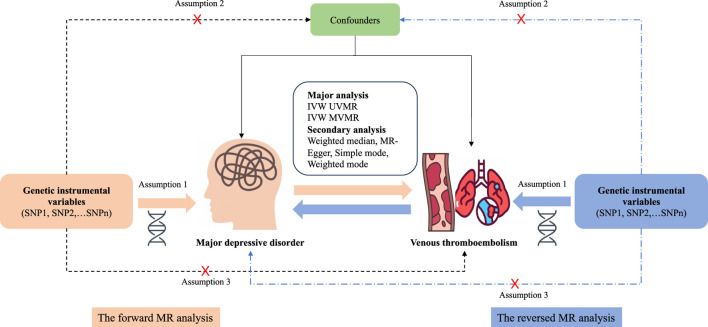
Overview and assumptions of the Mendelian randomization study design. Assumption 1: The instrumental variables should be closely related to the risk factor of interest; assumption 2: The instrumental variables should not be associated with potential confounders; and assumption 3: The instrumental variables should affect the risk of outcome only through risk factors and not through other alternative pathways. SNP, single-nucleotide polymorphisms; MDD, major depressive disorder; VTE, venous thromboembolism; PE, pulmonary embolism; DVT, deep venous thrombosis; IVW, inverse-variance weighting; UVMR, univariable Mendelian randomization; MVMR, multivariable Mendelian randomization.

This bidirectional two-sample MR analysis was based on the summary of genetic associations for different genome-wide association studies (GWAS) using single-nucleotide polymorphisms (SNPs) as IVs to investigate the causal associations between MDD and VTE. To gain reliable results, the effective IVs must satisfy three key assumptions during the MR analysis process: 1) the IVs must be strongly associated with the exposure; 2) IVs should not be associated with any confounders influencing the exposure and outcome association; and 3) IVs should be associated with the outcomes only *via* the exposures (Emdin et al., 2017). First, a forward MR analysis was performed to examine the associations between MDD and VTE. Next, a reverse MR analysis was performed to examine the associations of genetically determined VTE with MDD. The study design of MR analysis is shown in Figure 1.

### 2.2 Genetic data source of MDD and VTE

For our analyses, data regarding MDD and VTE were obtained from publicly available large-scale GWAS databases. The summary genetic associations of MDD statistics were obtained from the Psychiatric Genomics Consortium and UK Biobank, extracted from 170,756 cases and 329,443 controls based on European samples (Howard et al., 2019). To investigate the genetic associations with VTE, we extracted GWAS datasets from the FinnGen Biobank (Release 8, https://finngen.gitbook.io/documentation/v/r8/data-download). The latest version of the database was used for VTE (17,048 cases and 325,451 controls), PE (8,170 cases and 333,487 controls), and DVT (8,077 cases and 295,014 controls). Based on the International Classification of Diseases version 9, the definitions of VTE, DVT, and PE were developed. Detailed information on the GWAS in our study is presented in Supplementary Table S1.

### 2.3 Selection of IVs

From GWAS summary data of MDD and VTE, only SNPs strongly associated with exposure (*p* < 5 × 10^−8^) were extracted as genetic instruments. Linkage disequilibrium among SNPs defined by *r* ^2^ > 0.001 or clump distance <10,000 kb and those with higher *p* values were discarded, and the remaining SNPs were used as instruments. Next, the SNPs were then harmonized to ensure that the effect alleles were consistent in terms of both outcome and exposure data. Subsequently, we performed Steiger filtering to determine whether the directionality of a single SNP was correct and removed SNPs that were more strongly associated with outcome than with exposure (Hemani et al., 2017). The strength of IVs was examined using F-statistics; when the corresponding F statistic was >10, the instrument’s strength was deemed to be sufficient (Brion et al., 2013). We applied *m*R*n*d for power calculation (https://shiny.cnsgenomics.com/mRnd/), assuming a 5% type I error rate and power ≥80%. We used the PhenoScanner database to investigate the previously reported associations between instrument SNPs and potential confounding factors, and SNPs associated with potential confounders or outcome variables at genome-wide significance (*p* < 5 × 10^−8^) were removed (Kamat et al., 2019). The PhenoScanner database search revealed associations of instruments with education and body mass index (BMI) traits. Observational studies have also demonstrated that education and BMI are associated with the incidence of MDD and VTE (Zöller et al., 2012; Rahmani et al., 2020; Huet et al., 2021; Wickersham et al., 2021; de Wit et al., 2022). Therefore, we chose SNPs associated with education and BMI from GWAS for multivariable MR analysis. Details of the GWAS of risk cofounders are presented in Supplementary Table S2.

### 2.4 Statistical analyses

In our study, we tested the heterogeneity using Cochran’s Q test, and Cochran’s Q-derived *p* < 0.05 indicated heterogeneity. We applied inverse-variance weighting (IVW) as the primary analysis method to evaluate the relationship between MDD and VTE (Burgess et al., 2013). Furthermore, to assess the robustness of the results, other sensitivity analyses, including the weighted median (Bowden et al., 2016), MR-Egger regression (Bowden et al., 2015), weighted mode (Hartwig et al., 2017), simple mode (Zhu et al., 2022), and MR–pleiotropy residual sum and outlier (MR-PRESSO) (Verbanck et al., 2018) were conducted. The IVW method synthesizes the effect derived from each instrument by using the inverse variance as the weight and is applied on the assumption that all SNPs are valid instrumental variables and completely independent of each other (Burgess et al., 2013). The weighted median model can provide consistent estimates when ≥50% of the weights come from valid IVs (Bowden et al., 2016). In contrast, the MR-Egger method allows each IV to exhibit pleiotropy, and if the instrument strength is not related to these pleiotropic effects, the method is consistent (Bowden et al., 2015). The MR-Egger regression can inspect the horizontal pleiotropy through its intercept, and a *p*-value of >0.05 indicates no potential horizontal pleiotropy (Bowden et al., 2015). The MR-PRESSO method can detect the outliers and generate estimates after removing the outliers, which is designed to test the difference in the estimation before and after the outlier correction, with *p* < 0.05 of the distortion tests indicating a significant difference (Verbanck et al., 2018). To identify whether the association could be affected by a particular single SNP, we used the leave-one-out test to test the sensitivity of the results. Finally, a multivariable MR analysis was performed to assess the causal relationship between MDD and VTE, adjusting for education and BMI in our models to assess the effect of potential confounders. The multivariable MR simultaneously considers multiple exposure factors; it limits the corresponding effects of SNP exposure on the characteristics of other assumed risk factors and eliminates possible biases (Huang G. et al., 2023).

All statistical analysis was performed using R Software (version 4.1.2). TwoSampleMR (0.5.7), MR-PRESSO (1.0), and Mendelian Randomization (0.7.0) packages were used for the MR analysis. After Bonferroni correction, results with *p* < 0.05/3 (1 exposure × 3 outcomes) = 0.017 were considered statistically significant; estimates with a *p*-value between 0.017 and 0.05 were regarded as evidence suggesting an association in our study (Huang Y. et al., 2023).

## 3 Results

### 3.1 Basic information of IVs

The F-statistic of all included SNPs was >10, suggesting strong instruments (Supplementary Tables S3, S4). The MR-Steiger showed that the SNPs were more strongly associated with exposure than with outcome. For the MR statistical power, assuming a 5% type I error rate and power ≥80%, the power was low in both forward and reversed MR analysis (Supplementary Table S5).

### 3.2 Univariable MR analysis of MDD and VTE risk

**FIGURE 2:**
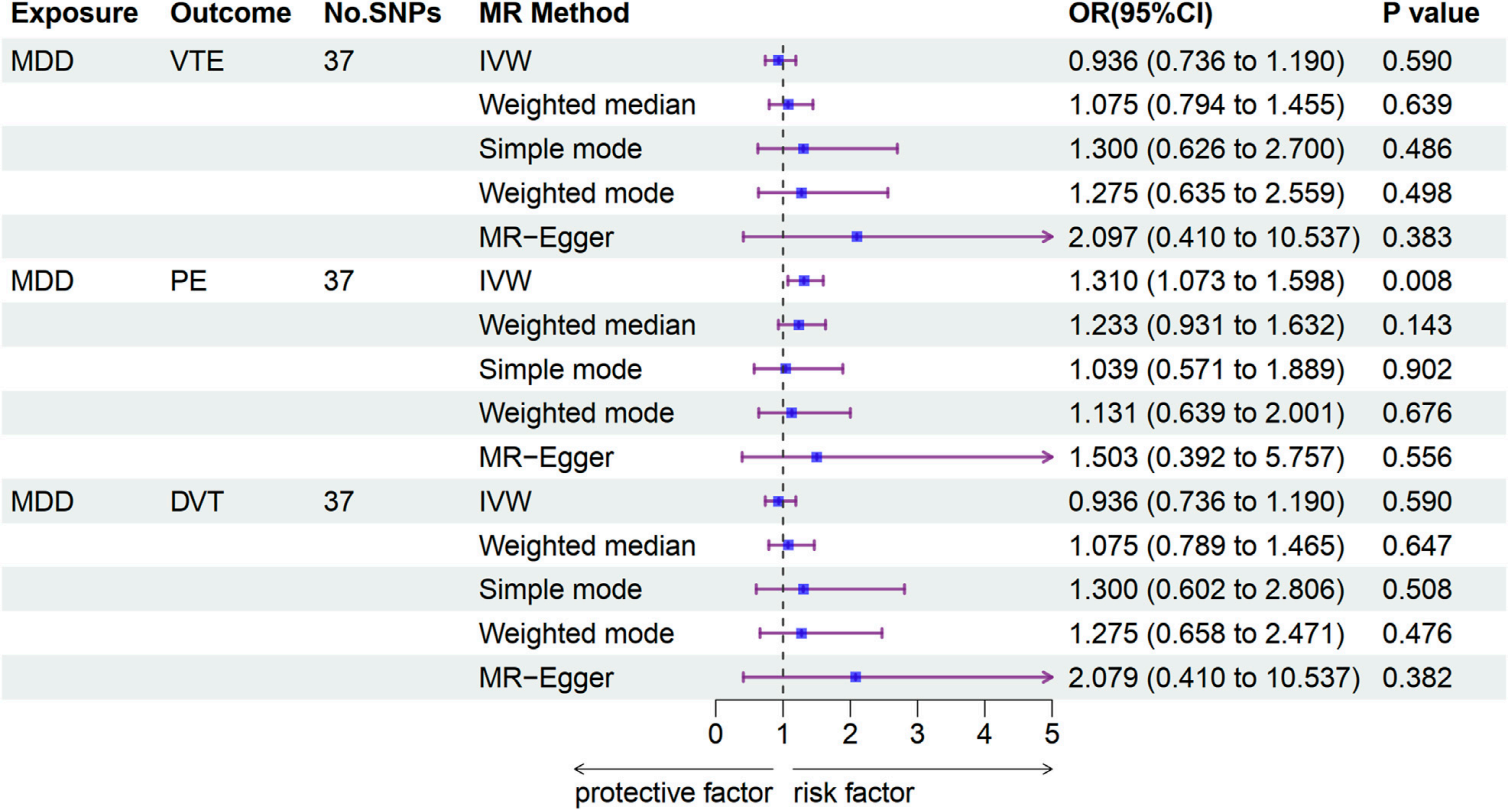
Associations of genetically predicted MDD with VTE development according to Mendelian randomization analyses. MDD, major depressive disorder; VTE, venous thromboembolism; PE, pulmonary embolism; DVT, deep venous thrombosis; OR, odds ratio; CI, confidence interval.

In the univariable MR analysis, independent SNPs (37 each) were selected and used as instruments in the MR analysis for the associations of MDD with VTE, PE, and DVT (Supplementary Table S3). The IVW results showed no significant causal association between MDD and VTE (odds ratio (OR): 0.936, 95% confidence interval (CI): 0.736–1.190, *p* = 0.590) or DVT risk (OR: 0.936, 95% CI: 0.736–1.190, *p* = 0.590); furthermore, we observed similar results by using weighted median, MR Egger, weighted mode, and simple mode. The IVW results suggested a significant causal association between MDD and PE risk (OR: 1.310, 95% CI: 1.073–1.598, *p* = 0.008); however, we observed different results by using weighted median, MR Egger, weighted mode, and simple mode (Figure 2 and Supplementary Table S6). No significant association was observed between MDD and VTE, PE, or DVT risk using MR-PRESSO (Supplementary Table S5). Cochran’s Q test supported the existence of significant heterogeneity between exposure and outcome (*P*-heterogeneity <0.05; Supplementary Table S6). The MR-Egger intercept provided no statistical evidence of directional horizontal pleiotropy (all *p* > 0.05, Supplementary Figures S1–S3). The leave-one-out analyses identified no individual IV, which largely affected the causal magnitude between MDD and the risk of VTE, PE, and DVT under the IVW model (Supplementary Figures S4–S6).

### 3.3 Multivariable MR analysis of MDD and VTE risk

After adjusting for potentially relevant polymorphisms related to education and BMI, the multivariable MR results showed evidence suggestive of an association between MDD and VTE risk (OR: 1.163, 95% CI: 1.004–1.346, *p* = 0.044). The likely reason for this is that the true effect was masked by confounding factors since the univariable analyses were not adjusted for them. After the relevant adjustments were performed, the effect became evident.

No significant causal association was observed between MDD and the risk of PE (OR: 1.197, 95% CI: 0.984–1.457, *p* = 0.072). This difference may be because education and BMI are more strongly correlated with PE and are potential risk factors for PE. When these two variables were considered, their stronger explanatory power for PE caused that of MDD for PE to be relatively weaker, resulting in a non-significant correlation between MDD and PE. No genetic causal associations were observed between MDD and the risk of DVT in multivariable analyses (OR: 0.930, 95% CI: 0.755–1.147, *p* = 0.499) (Table 1).

**TABLE 1:** Associations between MDD and VTE risk according to multivariable Mendelian randomization sensitivity analyses.

Exposure	Outcome	Methods	OR	95% CI	*p*-Value
MDD	VTE	MVMR_IVW	1.163	(1.004–1.346)	0.044
MDD	PE	MVMR_IVW	1.197	(0.984–1.457)	0.072
MDD	DVT	MVMR_IVW	0.930	(0.755–1.147)	0.499
VTE	MDD	MVMR_IVW	1.008	(0.984–1.033)	0.499
PE	MDD	MVMR_IVW	1.015	(0.992–1.039)	0.193
DVT	MDD	MVMR_IVW	0.996	(0.977–1.014)	0.640

MDD, major depressive disorder; VTE, venous thromboembolism; PE, pulmonary embolism; DVT, deep vein thrombosis; IVW, inverse-variance weighting; OR, odds ratio; CI, confidence interval; MVMR, multivariable Mendelian randomization.

### 3.4 Univariable MR analysis of VTE and MDD risk

**FIGURE 3:**
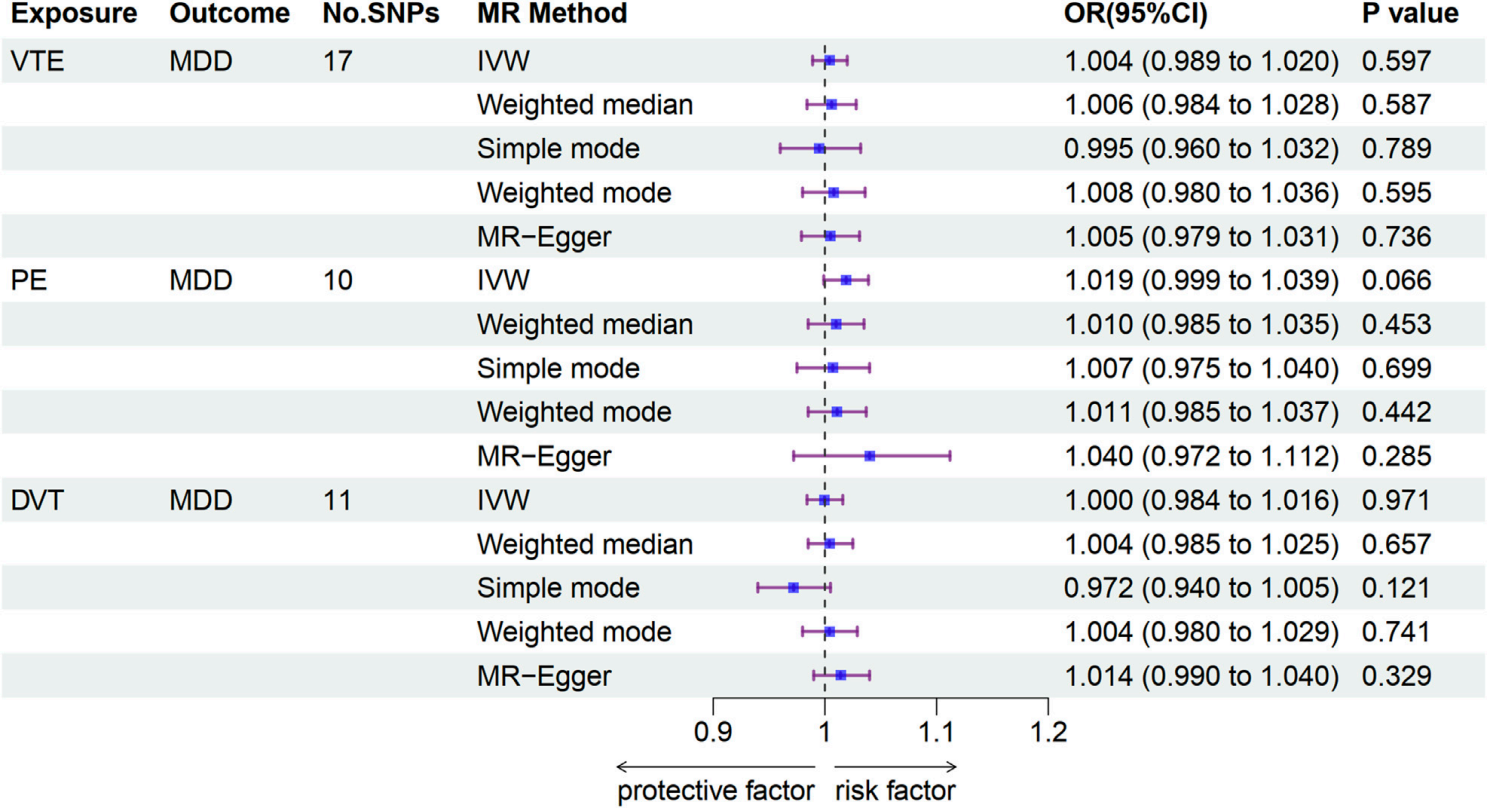
Associations of genetically predicted VTE with MDD according to Mendelian randomization analyses. MDD, major depressive disorder; VTE, venous thromboembolism; PE, pulmonary embolism; DVT, deep venous thrombosis; OR, odds ratio; CI, confidence interval.

A total of 17, 10, and 11 independent SNPs were used to explore the causal associations of VTE, PE, and DVT with the MDD risk, respectively (Supplementary Table S4). The IVW results suggested no significant associations between VTE (OR: 1.004, 95% CI: 0.989–1.020, *p* = 0.597), PE (OR: 1.019, 95% CI: 0.999–1.039, *p* = 0.066), or DVT (OR: 1.000, 95% CI: 0.984–1.016, *p* = 0.971) and MDD risk. We observed similar results by using weighted median, MR Egger, weighted mode, and simple mode (Figure 3 and Supplementary Table S7). Additionally, no significant association was observed between VTE, PE, or DVT and MDD risk using MR-PRESSO (Supplementary Table S5). Cochran’s Q test supported the existence of significant heterogeneity between exposure and outcome (*P*-heterogeneity <0.05; Supplementary Table S7). The MR-Egger intercept provided no existence of horizontal pleiotropy (all *p* > 0.05, Supplementary Figures S7–S9). The leave-one-out analyses showed that the MR analyses were stable and not affected by individual SNPs (Supplementary Figures S10–S12).

### 3.5 Multivariable MR analysis of VTE and MDD risk

The multivariable MR analysis results were consistent with the univariable MR analysis results. No significant causal association was observed between VTE (OR: 1.008, 95% CI: 0.984–1.033, *p* = 0.499), PE (OR: 1.015, 95% CI: 0.992–1.039, *p* = 0.193), or DVT (OR: 1.014, 95% CI: 0.977–1.014, *p* = 0.640) and MDD risk (Table 1).

## 4 Discussion

In this study, we performed a bidirectional two-sample MR analysis to investigate a genetically predicted causal association between MDD and VTE. Our findings provided suggestive evidence of the association between MDD and VTE risk. Furthermore, the reversed MR analysis found no genetic evidence of any causal association between VTE, PE, or DVT and MDD risk in either the univariable or the multivariable analyses.

We used BMI and education as confounders in our study. Previous studies have shown the correlation of BMI with depression and VTE. A representative psychiatric cohort study in the Netherlands showed that individuals with obesity at baseline had a significantly increased risk of developing any mood or anxiety disorder, even after adjusting for covariates compared to persons with a normal BMI (de Wit et al., 2022). Another observational study indicated that adiposity characteristics may help identify individuals at increased risk for neuropsychiatric comorbidity (Huet et al., 2021). A meta-analysis with 3,910,747 participants highlights obesity as a significant risk factor related to the incidence of VTE and PE (Rahmani et al., 2020). Additionally, the level of education was associated with depression and the incidence of VTE. A meta-analysis of 22 studies revealed that depression was associated with lower educational attainment (Wickersham et al., 2021). Furthermore, an epidemiological study from Sweden showed that individuals with high educational levels and those in several occupations requiring high levels of education had a lower risk of VTE, while those with low levels of education had an increased risk of VTE (Zöller et al., 2012).

### 4.1 MDD and VTE risk

Overall, we verified the relationship between MDD and VTE, which is consistent with several experimental studies. Recently, studies have shown that depression may increase the risk of VTE through genetic and biological mechanisms as well as behavioural mechanisms (Gold, 2015; Adelborg et al., 2017). Amadio et al. (Amadio et al., 2017) investigated the relationship between depression and VTE at a molecular level and found that it may be related to the presence of SNP variants on the gene encoding brain-derived neurotrophic factor, which increases susceptibility to depression while leading to concomitant hypercoagulability and platelet hyperreactivity in individuals. Moreover, the levels of numerous pro-inflammatory markers are higher in patients with depression than in the general population (van Aken et al., 2002; Reitsma and Rosendaal, 2004). Pro-inflammatory markers may reduce the activity of vasodilatory factors through a microvascular endothelium-dependent mechanism, inhibit endothelial nitric oxide synthase activity, and increase endothelin-1 secretion, leading to abnormal microvessel constriction, vasodilatory dysfunction, narrowing of the lumen, slowing of the blood flow, and thrombosis (Zi-xin and Yong-gui, 2021). Furthermore, acquired behavioural habits in patients with depression are associated with VTE. An epidemiological survey in Europe (Austin et al., 2013) showed that 73% of patients with depression had hypodynamic symptoms, including reduced energy and fatigue, which further increased the risk of thrombosis.

Several previous observational studies have demonstrated the association between depression and VTE, while others have shown opposite results (Austin et al., 2013; Lee et al., 2015; Parkin et al., 2017; Kunutsor et al., 2018; Kowal et al., 2020). A retrospective cohort study in Taiwan found that the risk of venous thrombosis was 1.38 times higher in patients with depression than in those without depression (Lee et al., 2015). In a prospective study, the risk of venous thrombosis was 1.6 times higher in patients with depression than in those without depression over a 12-year follow-up period (Austin et al., 2013). A systematic analysis of the risk of venous thrombosis in all psychiatric disorders collected since 1998 showed that the risk of venous thrombosis in patients with depression was 1.29 times higher than that in controls (Kowal et al., 2020). The systematic review and meta-analysis of observational studies published in 2018 included eight observational studies involving 960,113 non-overlapping participants and 9,027 VTE cases. Their results showed an increased risk of VTE in individuals with depression (RR = 1.31) compared with those without depression (Kunutsor et al., 2018). However, some studies showed inconsistent results. For example, a large prospective study involving women in the United Kingdom demonstrated that VTE risk was not significantly increased in women who reported being treated for depression or anxiety but did not use antidepressants or other psychotropic drugs (hazard ratio, 1.19) (Parkin et al., 2017).

Nevertheless, the precise pathophysiology underlying the association between MDD and VTE remains unknown. Therefore, our MR analysis was conducted to clarify the connection between them, and the results provide novel insights into the impact of MDD on VTE and potential avenues for prevention.

### 4.2 VTE and MDD risk

In our study, we observed no association between VTE and MDD risk, which differed from the results of previous studies.

Previous studies have shown that VTE may lead to depression through various psychosocial mechanisms. VTE is an acute, life-threatening event that causes deterioration in functioning, including pain, swelling, and reduced mobility, resulting in a reduced quality of life; additionally, an increased risk of bleeding due to the prolonged use of anticoagulants may lead to depression (Ghanima et al., 2018; Jørgensen et al., 2021). A 10-year population-cohort study involving more than 380,000 patients in Sweden showed that individuals diagnosed with VTE had a 2.35-fold increased risk of depression compared with the general population, and the risk estimates were moderately attenuated after adjusting for socioeconomic status and comorbidities (Jørgensen et al., 2023). However, the observed associations may be due to confounding factors such as education level, lifestyle, diet, or mobility problems, which are common features in some of these patients. In our study, we eliminated the confounders and found no association between VTE and MDD risk. To the best of our knowledge, this is the first comprehensive MR examination of the risks associated with venous thrombosis and depression.

### 4.3 Strengths and limitations

Our study has several strengths. First, according to the core principles of MR, this method can largely avoid the influence of reverse causality and confounding factors, which typically affect the results of conventional observational studies. Second, in this study, two-sample bidirectional MR was first applied to investigate the causal relationship between MDD and VTE, considering the large sample size and the comprehensive types of VTE. Third, regarding instrument selection, we had a strict selection threshold (*p* < 5 × 10^−8^) to reduce weak instrument bias. The F-statistics of all associations in our study exceeded 10, indicating the appropriate strength of the genetic instruments and the absence of weak instrument bias. Additionally, we used the multivariable MR methods to account for the potential pleiotropic associations between MDD and VTE.

However, our study has some limitations. First, to ensure homogeneity of genetic backgrounds, our study only included individuals of European descent, thereby limiting the generalizability of our results to other ethnic populations. Second, multivariable analysis could not overcome bias owing to pleiotropic effects from confounders other than education or BMI. Furthermore, because our findings may have had insufficient statistical power, a large amount of data from the GWAS databases is required to confirm the relationship between depression and different types of VTE.

## 5 Conclusion

Our MR analysis provided suggestive genetic evidence to support the causal association between MDD and VTE risk. Moreover, no causal associations were observed between VTE, PE, or DVT and MDD risk. Further validation of these associations and investigations of potential mechanisms are required.

## Data Availability

The datasets presented in this study can be found in online repositories. The names of the repository/repositories and accession number(s) can be found in the article/Supplementary Material.
