# Supplementary material for: Causal association between major depressive disorder and venous thromboembolism: a bidirectional mendelian randomization study
Source: Front Genet. 2024 Jun 25;15:1383333. doi: 10.3389/fgene.2024.1383333 (PMC11231919; doi:10.3389/fgene.2024.1383333)
Supplement: Supplementary file 1 [file Table1.DOCX]

**Causal association between major depressive disorder and venous thromboembolism: A bidirectional Mendelian randomization study**

Hong-Yan Li^1^, Li-Hong Wang^2^, Jing Wang^1^, Yong-Bo Wang^3^, Hai-Shan Wang^4^

**Supplementary Tables and Figures**

**Supplementary Table 1.** Study details for the genome-wide association studies of exposures and outcomes

**Supplementary Table 2.** Details of GWAS used for risk cofounders

**Supplementary Table 3.** Summary information on the exposure instruments used in the Mendelian randomization analyses

**Supplementary Table 4.** Summary information on the exposure instruments of VTE used in the Mendelian randomization analyses

**Supplementary Table 5.** The results of MR-PRESSO and the MR statistical power

**Supplementary Table 6.** Associations of genetically determined MDD with VTE

**Supplementary Table 7.** Associations of genetically determined VTE with MDD

**Supplementary Figure 1.** Scatterplots comparing the strength of the SNP-exposure (MDD) and SNP-VTE associations

**Supplementary Figure 2.** Scatterplots comparing the strength of the SNP-exposure (MDD) and SNP-PE associations

**Supplementary Figure 3.** Scatterplots comparing the strength of the SNP-exposure (MDD) and SNP-DVT associations

**Supplementary Figure 4.** Leave-one-out meta-analysis. The leave-one-out plot visualized how the causal estimates (point with horizontal line) for the effect of MDD on VTE were influenced by the removal of single variant

**Supplementary Figure 5.** Leave-one-out meta-analysis. The leave-one-out plot visualized how the causal estimates (point with horizontal line) for the effect of MDD on PE were influenced by the removal of single variant

**Supplementary Figure 6.** Leave-one-out meta-analysis. The leave-one-out plot visualized how the causal estimates (point with horizontal line) for the effect of MDD on DVT were influenced by the removal of single variant

**Supplementary Figure 7.** Scatterplots comparing the strength of the SNP-exposure (VTE) and SNP-MDD associations

**Supplementary Figure 8.** Scatterplots comparing the strength of the SNP-exposure (PE) and SNP-MDD associations

**Supplementary Figure 9.** Scatterplots comparing the strength of the SNP-exposure (DVT) and SNP-MDD associations

**Supplementary Figure 10.** Leave-one-out meta-analysis. The leave-one-out plot visualized how the causal estimates (point with horizontal line) for the effect of VTE on MDD were influenced by the removal of single variant

**Supplementary Figure 11.** Leave-one-out meta-analysis. The leave-one-out plot visualized how the causal estimates (point with horizontal line) for the effect of PE on MDD were influenced by the removal of single variant

**Supplementary Figure 12.** Leave-one-out meta-analysis. The leave-one-out plot visualized how the causal estimates (point with horizontal line) for the effect of DVT on MDD were influenced by the removal of single variant

**Supplementary Table 1.** Study details for the genome-wide association studies of exposures and outcomes

| **Exposure or outcome** | **Study or consortium** | **Sample size** | **Case** | **Control** | **Ethnicity** | **Year of release** |
| --- | --- | --- | --- | --- | --- | --- |
| MDD | UKB and PGC | 500199 | 170756 | 329443 | European | 2019 |
| VTE | FinnGen | 342499 | 17048 | 325451 | European | 2022 |
| PE | FinnGen | 341657 | 8170 | 333487 | European | 2022 |
| DVT | FinnGen | 303118 | 8077 | 295041 | European | 2022 |

MDD, major depressive disorder; VTE, venous thromboembolism; PE, pulmonary embolism; DVT, deep vein thrombosis; UKB, United Kingdom Biobank; PGC, Psychiatric Genomics Consortium.

**Supplementary Table 2.** Details of GWAS used for risk cofounders

| **covariates** | **Sample size** | **Case** | **Control** | **Ethnicity** | **Year of release** |
| --- | --- | --- | --- | --- | --- |
| Body mass index | 681275 | / | / | European | 2019 |
| Qualifications: college or university degree | 458079 | 148722 | 309357 | European | 2019 |

**Supplementary Table 3.** Summary information on the exposure instruments used in the Mendelian randomization analyses

| **Exposure** | **Outcome** | **SNP** | **Chr** | **Effect allele** | **Other allele** | **eaf.exposure** | **beta.exposure** | **SE.exposure** | **P value.exposure** | **beta.outcome** | **SE.outcome** | **P value.outcome** | **FSTAT** |
| --- | --- | --- | --- | --- | --- | --- | --- | --- | --- | --- | --- | --- | --- |
| MDD | VTE | rs1021363 | 10 | G | A | 0.6434 | -0.03 | 0.0045 | 2.28718E-11 | -0.0229958 | 0.0180802 | 0.203417 | 44.44426674 |
| MDD | VTE | rs10913112 | 1 | T | C | 0.378 | -0.0262 | 0.0045 | 4.52501E-09 | -0.0206606 | 0.0166856 | 0.21563 | 33.89813607 |
| MDD | VTE | rs12919291 | 16 | C | G | 0.1884 | 0.0327 | 0.0055 | 3.092E-09 | -0.0507056 | 0.0222932 | 0.0229372 | 35.34828841 |
| MDD | VTE | rs12967143 | 18 | C | G | 0.7012 | -0.0345 | 0.0047 | 2.52697E-13 | -0.0297262 | 0.0185004 | 0.1081 | 53.88163155 |
| MDD | VTE | rs13037326 | 20 | T | C | 0.2597 | 0.031 | 0.0049 | 2.398E-10 | -0.0126579 | 0.0178057 | 0.477152 | 40.02482955 |
| MDD | VTE | rs1367635 | 18 | C | T | 0.5148 | 0.0253 | 0.0043 | 4.35402E-09 | 0.0258785 | 0.0161354 | 0.10875 | 34.61803357 |
| MDD | VTE | rs150186873 | 6 | C | A | 0.0327 | 0.0704 | 0.012 | 4.51305E-09 | 0.0166101 | 0.0603861 | 0.783267 | 34.41764016 |
| MDD | VTE | rs150346963 | 7 | T | C | 0.4118 | 0.0283 | 0.0044 | 1.15699E-10 | 0.00986596 | 0.0161835 | 0.542107 | 41.36811972 |
| MDD | VTE | rs17641524 | 1 | T | C | 0.2101 | -0.03 | 0.0053 | 1.502E-08 | 0.0228294 | 0.0227137 | 0.314852 | 32.03974373 |
| MDD | VTE | rs1931388 | 9 | G | A | 0.4042 | -0.0295 | 0.0044 | 1.67996E-11 | 0.0215494 | 0.0162769 | 0.185529 | 44.95075002 |
| MDD | VTE | rs1950829 | 14 | G | A | 0.5173 | -0.0297 | 0.0043 | 4.73805E-12 | -0.00436867 | 0.0160839 | 0.785916 | 47.706137 |
| MDD | VTE | rs198457 | 11 | T | C | 0.1886 | -0.0315 | 0.0056 | 1.89998E-08 | 0.0387285 | 0.0268909 | 0.149809 | 31.64049849 |
| MDD | VTE | rs2111592 | 2 | A | G | 0.3141 | 0.0263 | 0.0046 | 1.34999E-08 | -0.0185648 | 0.0168926 | 0.271772 | 32.68843262 |
| MDD | VTE | rs2214123 | 6 | G | A | 0.6466 | -0.0261 | 0.0045 | 8.55598E-09 | 0.0122976 | 0.0166864 | 0.461132 | 33.63986549 |
| MDD | VTE | rs2418449 | 9 | C | T | 0.281 | -0.0281 | 0.0048 | 4.24502E-09 | 0.0144118 | 0.0172967 | 0.404724 | 34.27113033 |
| MDD | VTE | rs247910 | 5 | G | A | 0.457 | 0.0237 | 0.0043 | 4.71205E-08 | -0.0269365 | 0.0161328 | 0.0949839 | 30.37792072 |
| MDD | VTE | rs2522831 | 7 | C | T | 0.4739 | 0.024 | 0.0043 | 2.113E-08 | 0.0208852 | 0.016082 | 0.194055 | 31.15184948 |
| MDD | VTE | rs28541419 | 15 | G | C | 0.2308 | -0.0292 | 0.0052 | 1.75598E-08 | -0.0124033 | 0.0215951 | 0.565726 | 31.5324183 |
| MDD | VTE | rs354155 | 1 | C | G | 0.0923 | -0.0449 | 0.0075 | 1.75102E-09 | -0.00965967 | 0.0221126 | 0.662227 | 35.84003447 |
| MDD | VTE | rs3807865 | 7 | A | G | 0.4105 | 0.031 | 0.0044 | 1.09295E-12 | -0.027869 | 0.0166808 | 0.0947764 | 49.63823128 |
| MDD | VTE | rs4141983 | 1 | C | T | 0.326 | -0.0264 | 0.0046 | 9.69192E-09 | -0.0103375 | 0.0170112 | 0.543397 | 32.93748645 |
| MDD | VTE | rs4497414 | 11 | C | T | 0.44 | 0.0291 | 0.0044 | 2.92685E-11 | -0.0244158 | 0.016303 | 0.13423 | 43.74001106 |
| MDD | VTE | rs4936276 | 11 | C | G | 0.622 | 0.0278 | 0.0044 | 3.57001E-10 | 0.0137294 | 0.0197965 | 0.48798 | 39.91926187 |
| MDD | VTE | rs508502 | 13 | T | C | 0.2992 | -0.0264 | 0.0048 | 3.55599E-08 | 0.0200137 | 0.0171009 | 0.241868 | 30.24987905 |
| MDD | VTE | rs59082935 | 7 | T | C | 0.1342 | 0.0363 | 0.0066 | 3.071E-08 | 0.0493455 | 0.0225024 | 0.0283146 | 30.24987905 |
| MDD | VTE | rs61914045 | 12 | A | G | 0.2034 | 0.0309 | 0.0054 | 7.96398E-09 | -0.00298073 | 0.0206619 | 0.885294 | 32.74369624 |
| MDD | VTE | rs62535714 | 9 | A | G | 0.1639 | 0.0339 | 0.0058 | 4.68598E-09 | -0.0471685 | 0.0187216 | 0.0117533 | 34.16187292 |
| MDD | VTE | rs66511648 | 3 | C | T | 0.284 | 0.0297 | 0.0048 | 6.03004E-10 | -0.00634024 | 0.0195734 | 0.745997 | 38.28500317 |
| MDD | VTE | rs7152906 | 14 | C | T | 0.5196 | 0.0258 | 0.0043 | 1.87301E-09 | 0.000635115 | 0.0160908 | 0.968515 | 35.99985606 |
| MDD | VTE | rs7241572 | 18 | A | G | 0.2047 | 0.0323 | 0.0054 | 2.43299E-09 | 0.0163504 | 0.0196453 | 0.40525 | 35.77797766 |
| MDD | VTE | rs7538938 | 1 | C | T | 0.5599 | 0.0251 | 0.0043 | 7.28903E-09 | -0.0251262 | 0.0161676 | 0.120158 | 34.0728762 |
| MDD | VTE | rs7551758 | 1 | G | T | 0.5329 | 0.0283 | 0.0043 | 5.1074E-11 | 0.00523791 | 0.0161164 | 0.745176 | 43.31459155 |
| MDD | VTE | rs76954012 | 3 | A | T | 0.0931 | 0.0412 | 0.0074 | 2.41002E-08 | 0.0571439 | 0.034438 | 0.097051 | 30.99768468 |
| MDD | VTE | rs7725715 | 5 | A | G | 0.5343 | 0.029 | 0.0043 | 1.60694E-11 | 0.000259702 | 0.0165443 | 0.987476 | 45.48386357 |
| MDD | VTE | rs9364755 | 6 | G | A | 0.2262 | 0.0283 | 0.0051 | 3.48602E-08 | 0.00594948 | 0.0179661 | 0.740531 | 30.79149549 |
| MDD | VTE | rs9529218 | 13 | T | C | 0.2031 | -0.034 | 0.0054 | 2.231E-10 | -0.0126417 | 0.0179273 | 0.480708 | 39.64318854 |
| MDD | VTE | rs9536381 | 13 | T | C | 0.3259 | 0.0255 | 0.0046 | 2.61698E-08 | -0.00898127 | 0.0170054 | 0.5974 | 30.73002836 |
| MDD | PE | rs1021363 | 10 | G | A | 0.6434 | -0.03 | 0.0045 | 2.28718E-11 | -0.0300474 | 0.0179276 | 0.0937303 | 44.44426674 |
| MDD | PE | rs10913112 | 1 | T | C | 0.378 | -0.0262 | 0.0045 | 4.52501E-09 | 0.0117301 | 0.0165471 | 0.478391 | 33.89813607 |
| MDD | PE | rs12919291 | 16 | C | G | 0.1884 | 0.0327 | 0.0055 | 3.092E-09 | -0.00224335 | 0.0221087 | 0.919178 | 35.34828841 |
| MDD | PE | rs12967143 | 18 | C | G | 0.7012 | -0.0345 | 0.0047 | 2.52697E-13 | -0.0151994 | 0.0183671 | 0.407936 | 53.88163155 |
| MDD | PE | rs13037326 | 20 | T | C | 0.2597 | 0.031 | 0.0049 | 2.398E-10 | -0.00746182 | 0.0177044 | 0.673415 | 40.02482955 |
| MDD | PE | rs1367635 | 18 | C | T | 0.5148 | 0.0253 | 0.0043 | 4.35402E-09 | 0.0443693 | 0.0159885 | 0.00551887 | 34.61803357 |
| MDD | PE | rs150186873 | 6 | C | A | 0.0327 | 0.0704 | 0.012 | 4.51305E-09 | 0.0713482 | 0.0600974 | 0.235145 | 34.41764016 |
| MDD | PE | rs150346963 | 7 | T | C | 0.4118 | 0.0283 | 0.0044 | 1.15699E-10 | 0.00753484 | 0.0160471 | 0.638681 | 41.36811972 |
| MDD | PE | rs17641524 | 1 | T | C | 0.2101 | -0.03 | 0.0053 | 1.502E-08 | 0.00927429 | 0.0225122 | 0.680363 | 32.03974373 |
| MDD | PE | rs1931388 | 9 | G | A | 0.4042 | -0.0295 | 0.0044 | 1.67996E-11 | 0.00688598 | 0.01617 | 0.670218 | 44.95075002 |
| MDD | PE | rs1950829 | 14 | G | A | 0.5173 | -0.0297 | 0.0043 | 4.73805E-12 | -0.0148074 | 0.0159586 | 0.353476 | 47.706137 |
| MDD | PE | rs198457 | 11 | T | C | 0.1886 | -0.0315 | 0.0056 | 1.89998E-08 | 0.0115009 | 0.0266167 | 0.665673 | 31.64049849 |
| MDD | PE | rs2111592 | 2 | A | G | 0.3141 | 0.0263 | 0.0046 | 1.34999E-08 | 0.0236713 | 0.0167647 | 0.157958 | 32.68843262 |
| MDD | PE | rs2214123 | 6 | G | A | 0.6466 | -0.0261 | 0.0045 | 8.55598E-09 | 0.00379576 | 0.0165605 | 0.818709 | 33.63986549 |
| MDD | PE | rs2418449 | 9 | C | T | 0.281 | -0.0281 | 0.0048 | 4.24502E-09 | -0.00267694 | 0.0171484 | 0.875951 | 34.27113033 |
| MDD | PE | rs247910 | 5 | G | A | 0.457 | 0.0237 | 0.0043 | 4.71205E-08 | -0.0323947 | 0.0160174 | 0.0431271 | 30.37792072 |
| MDD | PE | rs2522831 | 7 | C | T | 0.4739 | 0.024 | 0.0043 | 2.113E-08 | 0.0212541 | 0.0159555 | 0.182832 | 31.15184948 |
| MDD | PE | rs28541419 | 15 | G | C | 0.2308 | -0.0292 | 0.0052 | 1.75598E-08 | -0.0318644 | 0.0214284 | 0.137011 | 31.5324183 |
| MDD | PE | rs354155 | 1 | C | G | 0.0923 | -0.0449 | 0.0075 | 1.75102E-09 | -0.0432203 | 0.021958 | 0.049032 | 35.84003447 |
| MDD | PE | rs3807865 | 7 | A | G | 0.4105 | 0.031 | 0.0044 | 1.09295E-12 | 0.0210124 | 0.0165437 | 0.204045 | 49.63823128 |
| MDD | PE | rs4141983 | 1 | C | T | 0.326 | -0.0264 | 0.0046 | 9.69192E-09 | -0.00251597 | 0.0168846 | 0.881546 | 32.93748645 |
| MDD | PE | rs4497414 | 11 | C | T | 0.44 | 0.0291 | 0.0044 | 2.92685E-11 | 0.00236165 | 0.0161734 | 0.883905 | 43.74001106 |
| MDD | PE | rs4936276 | 11 | C | G | 0.622 | 0.0278 | 0.0044 | 3.57001E-10 | 0.0211536 | 0.0196306 | 0.281219 | 39.91926187 |
| MDD | PE | rs508502 | 13 | T | C | 0.2992 | -0.0264 | 0.0048 | 3.55599E-08 | -0.0185775 | 0.0169775 | 0.273848 | 30.24987905 |
| MDD | PE | rs59082935 | 7 | T | C | 0.1342 | 0.0363 | 0.0066 | 3.071E-08 | 0.014588 | 0.0223501 | 0.513948 | 30.24987905 |
| MDD | PE | rs61914045 | 12 | A | G | 0.2034 | 0.0309 | 0.0054 | 7.96398E-09 | -0.0178929 | 0.0205294 | 0.383439 | 32.74369624 |
| MDD | PE | rs62535714 | 9 | A | G | 0.1639 | 0.0339 | 0.0058 | 4.68598E-09 | 0.00251343 | 0.018585 | 0.892422 | 34.16187292 |
| MDD | PE | rs66511648 | 3 | C | T | 0.284 | 0.0297 | 0.0048 | 6.03004E-10 | -0.0100643 | 0.0194196 | 0.604281 | 38.28500317 |
| MDD | PE | rs7152906 | 14 | C | T | 0.5196 | 0.0258 | 0.0043 | 1.87301E-09 | 0.0293233 | 0.015964 | 0.0662338 | 35.99985606 |
| MDD | PE | rs7241572 | 18 | A | G | 0.2047 | 0.0323 | 0.0054 | 2.43299E-09 | -0.00236856 | 0.019513 | 0.903387 | 35.77797766 |
| MDD | PE | rs7538938 | 1 | C | T | 0.5599 | 0.0251 | 0.0043 | 7.28903E-09 | 0.0107664 | 0.0160411 | 0.502109 | 34.0728762 |
| MDD | PE | rs7551758 | 1 | G | T | 0.5329 | 0.0283 | 0.0043 | 5.1074E-11 | 0.0143163 | 0.0159922 | 0.370676 | 43.31459155 |
| MDD | PE | rs76954012 | 3 | A | T | 0.0931 | 0.0412 | 0.0074 | 2.41002E-08 | -0.0444453 | 0.0341699 | 0.193355 | 30.99768468 |
| MDD | PE | rs7725715 | 5 | A | G | 0.5343 | 0.029 | 0.0043 | 1.60694E-11 | -0.00920809 | 0.0164189 | 0.574918 | 45.48386357 |
| MDD | PE | rs9364755 | 6 | G | A | 0.2262 | 0.0283 | 0.0051 | 3.48602E-08 | 0.0022399 | 0.0178445 | 0.900109 | 30.79149549 |
| MDD | PE | rs9529218 | 13 | T | C | 0.2031 | -0.034 | 0.0054 | 2.231E-10 | -0.00641554 | 0.0177743 | 0.71814 | 39.64318854 |
| MDD | PE | rs9536381 | 13 | T | C | 0.3259 | 0.0255 | 0.0046 | 2.61698E-08 | 0.0135996 | 0.0168629 | 0.419966 | 30.73002836 |
| MDD | DVT | rs1021363 | 10 | G | A | 0.6434 | -0.03 | 0.0045 | 2.28718E-11 | -0.0229958 | 0.0180802 | 0.203417 | 44.44426674 |
| MDD | DVT | rs10913112 | 1 | T | C | 0.378 | -0.0262 | 0.0045 | 4.52501E-09 | -0.0206606 | 0.0166856 | 0.21563 | 33.89813607 |
| MDD | DVT | rs12919291 | 16 | C | G | 0.1884 | 0.0327 | 0.0055 | 3.092E-09 | -0.0507056 | 0.0222932 | 0.0229372 | 35.34828841 |
| MDD | DVT | rs12967143 | 18 | C | G | 0.7012 | -0.0345 | 0.0047 | 2.52697E-13 | -0.0297262 | 0.0185004 | 0.1081 | 53.88163155 |
| MDD | DVT | rs13037326 | 20 | T | C | 0.2597 | 0.031 | 0.0049 | 2.398E-10 | -0.0126579 | 0.0178057 | 0.477152 | 40.02482955 |
| MDD | DVT | rs1367635 | 18 | C | T | 0.5148 | 0.0253 | 0.0043 | 4.35402E-09 | 0.0258785 | 0.0161354 | 0.10875 | 34.61803357 |
| MDD | DVT | rs150186873 | 6 | C | A | 0.0327 | 0.0704 | 0.012 | 4.51305E-09 | 0.0166101 | 0.0603861 | 0.783267 | 34.41764016 |
| MDD | DVT | rs150346963 | 7 | T | C | 0.4118 | 0.0283 | 0.0044 | 1.15699E-10 | 0.00986596 | 0.0161835 | 0.542107 | 41.36811972 |
| MDD | DVT | rs17641524 | 1 | T | C | 0.2101 | -0.03 | 0.0053 | 1.502E-08 | 0.0228294 | 0.0227137 | 0.314852 | 32.03974373 |
| MDD | DVT | rs1931388 | 9 | G | A | 0.4042 | -0.0295 | 0.0044 | 1.67996E-11 | 0.0215494 | 0.0162769 | 0.185529 | 44.95075002 |
| MDD | DVT | rs1950829 | 14 | G | A | 0.5173 | -0.0297 | 0.0043 | 4.73805E-12 | -0.00436867 | 0.0160839 | 0.785916 | 47.706137 |
| MDD | DVT | rs198457 | 11 | T | C | 0.1886 | -0.0315 | 0.0056 | 1.89998E-08 | 0.0387285 | 0.0268909 | 0.149809 | 31.64049849 |
| MDD | DVT | rs2111592 | 2 | A | G | 0.3141 | 0.0263 | 0.0046 | 1.34999E-08 | -0.0185648 | 0.0168926 | 0.271772 | 32.68843262 |
| MDD | DVT | rs2214123 | 6 | G | A | 0.6466 | -0.0261 | 0.0045 | 8.55598E-09 | 0.0122976 | 0.0166864 | 0.461132 | 33.63986549 |
| MDD | DVT | rs2418449 | 9 | C | T | 0.281 | -0.0281 | 0.0048 | 4.24502E-09 | 0.0144118 | 0.0172967 | 0.404724 | 34.27113033 |
| MDD | DVT | rs247910 | 5 | G | A | 0.457 | 0.0237 | 0.0043 | 4.71205E-08 | -0.0269365 | 0.0161328 | 0.0949839 | 30.37792072 |
| MDD | DVT | rs2522831 | 7 | C | T | 0.4739 | 0.024 | 0.0043 | 2.113E-08 | 0.0208852 | 0.016082 | 0.194055 | 31.15184948 |
| MDD | DVT | rs28541419 | 15 | G | C | 0.2308 | -0.0292 | 0.0052 | 1.75598E-08 | -0.0124033 | 0.0215951 | 0.565726 | 31.5324183 |
| MDD | DVT | rs354155 | 1 | C | G | 0.0923 | -0.0449 | 0.0075 | 1.75102E-09 | -0.00965967 | 0.0221126 | 0.662227 | 35.84003447 |
| MDD | DVT | rs3807865 | 7 | A | G | 0.4105 | 0.031 | 0.0044 | 1.09295E-12 | -0.027869 | 0.0166808 | 0.0947764 | 49.63823128 |
| MDD | DVT | rs4141983 | 1 | C | T | 0.326 | -0.0264 | 0.0046 | 9.69192E-09 | -0.0103375 | 0.0170112 | 0.543397 | 32.93748645 |
| MDD | DVT | rs4497414 | 11 | C | T | 0.44 | 0.0291 | 0.0044 | 2.92685E-11 | -0.0244158 | 0.016303 | 0.13423 | 43.74001106 |
| MDD | DVT | rs4936276 | 11 | C | G | 0.622 | 0.0278 | 0.0044 | 3.57001E-10 | 0.0137294 | 0.0197965 | 0.48798 | 39.91926187 |
| MDD | DVT | rs508502 | 13 | T | C | 0.2992 | -0.0264 | 0.0048 | 3.55599E-08 | 0.0200137 | 0.0171009 | 0.241868 | 30.24987905 |
| MDD | DVT | rs59082935 | 7 | T | C | 0.1342 | 0.0363 | 0.0066 | 3.071E-08 | 0.0493455 | 0.0225024 | 0.0283146 | 30.24987905 |
| MDD | DVT | rs61914045 | 12 | A | G | 0.2034 | 0.0309 | 0.0054 | 7.96398E-09 | -0.00298073 | 0.0206619 | 0.885294 | 32.74369624 |
| MDD | DVT | rs62535714 | 9 | A | G | 0.1639 | 0.0339 | 0.0058 | 4.68598E-09 | -0.0471685 | 0.0187216 | 0.0117533 | 34.16187292 |
| MDD | DVT | rs66511648 | 3 | C | T | 0.284 | 0.0297 | 0.0048 | 6.03004E-10 | -0.00634024 | 0.0195734 | 0.745997 | 38.28500317 |
| MDD | DVT | rs7152906 | 14 | C | T | 0.5196 | 0.0258 | 0.0043 | 1.87301E-09 | 0.000635115 | 0.0160908 | 0.968515 | 35.99985606 |
| MDD | DVT | rs7241572 | 18 | A | G | 0.2047 | 0.0323 | 0.0054 | 2.43299E-09 | 0.0163504 | 0.0196453 | 0.40525 | 35.77797766 |
| MDD | DVT | rs7538938 | 1 | C | T | 0.5599 | 0.0251 | 0.0043 | 7.28903E-09 | -0.0251262 | 0.0161676 | 0.120158 | 34.0728762 |
| MDD | DVT | rs7551758 | 1 | G | T | 0.5329 | 0.0283 | 0.0043 | 5.1074E-11 | 0.00523791 | 0.0161164 | 0.745176 | 43.31459155 |
| MDD | DVT | rs76954012 | 3 | A | T | 0.0931 | 0.0412 | 0.0074 | 2.41002E-08 | 0.0571439 | 0.034438 | 0.097051 | 30.99768468 |
| MDD | DVT | rs7725715 | 5 | A | G | 0.5343 | 0.029 | 0.0043 | 1.60694E-11 | 0.000259702 | 0.0165443 | 0.987476 | 45.48386357 |
| MDD | DVT | rs9364755 | 6 | G | A | 0.2262 | 0.0283 | 0.0051 | 3.48602E-08 | 0.00594948 | 0.0179661 | 0.740531 | 30.79149549 |
| MDD | DVT | rs9529218 | 13 | T | C | 0.2031 | -0.034 | 0.0054 | 2.231E-10 | -0.0126417 | 0.0179273 | 0.480708 | 39.64318854 |
| MDD | DVT | rs9536381 | 13 | T | C | 0.3259 | 0.0255 | 0.0046 | 2.61698E-08 | -0.00898127 | 0.0170054 | 0.5974 | 30.73002836 |

MDD, major depressive disorder; VTE, venous thromboembolism; PE, pulmonary embolism; DVT, deep vein thrombosis.

**Supplementary Table 4.** Summary information on the exposure instruments of VTE used in the Mendelian randomization analyses

| **Exposure** | **Outcome** | **SNP** | **Chr** | **Effect allele** | **Other allele** | **eaf.exposure** | **beta.exposure** | **SE.exposure** | **P value.exposure** | **beta.outcome** | **SE.outcome** | **P value.outcome** | **FSTAT** |
| --- | --- | --- | --- | --- | --- | --- | --- | --- | --- | --- | --- | --- | --- |
| VTE | MDD | rs113079063 | 1 | T | G | 0.00902475 | 0.368099 | 0.0539898 | 9.23634E-12 | -0.0076 | 0.0138 | 0.581 | 46.4838261 |
| VTE | MDD | rs114026832 | 1 | A | C | 0.00198331 | 0.73884 | 0.106302 | 3.6425E-12 | 0.0199 | 0.0254 | 0.4325 | 48.3074328 |
| VTE | MDD | rs116997538 | 12 | T | C | 0.0159053 | 0.41254 | 0.0409674 | 7.50067E-24 | -0.0081 | 0.021 | 0.7013 | 101.403128 |
| VTE | MDD | rs12054563 | 4 | G | A | 0.108463 | -0.12994 | 0.0187619 | 4.33711E-12 | -0.0016 | 0.0106 | 0.8815 | 47.9654056 |
| VTE | MDD | rs12115818 | 9 | A | G | 0.0347107 | 0.177484 | 0.0298055 | 2.60465E-09 | -0.0034 | 0.0146 | 0.8145 | 35.4586031 |
| VTE | MDD | rs2066865 | 4 | A | G | 0.301778 | 0.193519 | 0.011934 | 3.90032E-59 | 0.0018 | 0.0051 | 0.7166 | 262.948795 |
| VTE | MDD | rs3756011 | 4 | A | C | 0.429138 | 0.197125 | 0.0112264 | 5.06991E-69 | 0.0046 | 0.0044 | 0.2989 | 308.317672 |
| VTE | MDD | rs57328376 | 14 | G | A | 0.3633 | 0.0712874 | 0.0116232 | 8.61192E-10 | 0.0047 | 0.0047 | 0.3175 | 37.6157876 |
| VTE | MDD | rs576123 | 9 | T | C | 0.563591 | -0.243055 | 0.0111707 | 5.78096E-105 | 0.0077 | 0.0046 | 0.0953191 | 473.41757 |
| VTE | MDD | rs6025 | 1 | T | C | 0.0199454 | 0.855164 | 0.0319648 | 1.1298E-157 | 0.0048 | 0.0139 | 0.7326 | 715.732713 |
| VTE | MDD | rs60681578 | 9 | C | A | 0.154058 | -0.123004 | 0.0159591 | 1.28322E-14 | 0.005 | 0.0063 | 0.4326 | 59.404276 |
| VTE | MDD | rs6132582 | 20 | G | A | 0.160626 | -0.0975103 | 0.0156479 | 4.6186E-10 | -0.0043 | 0.0062 | 0.4883 | 38.8315648 |
| VTE | MDD | rs62350309 | 4 | G | A | 0.102409 | -0.166576 | 0.0192747 | 5.51442E-18 | 0.0065 | 0.01 | 0.5134 | 74.6871064 |
| VTE | MDD | rs628094 | 9 | A | G | 0.682154 | 0.0856984 | 0.0121778 | 1.96065E-12 | -0.006 | 0.0049 | 0.2167 | 49.5226394 |
| VTE | MDD | rs78807356 | 11 | T | G | 0.00652177 | 0.5343 | 0.0602042 | 7.00487E-19 | 0.0267 | 0.0135 | 0.0482203 | 78.761286 |
| VTE | MDD | rs80137017 | 10 | T | C | 0.107697 | -0.205493 | 0.0189484 | 2.1096E-27 | 0 | 0.0065 | 0.9997 | 117.610205 |
| VTE | MDD | rs9820410 | 3 | A | G | 0.369204 | -0.0667523 | 0.0118689 | 1.86432E-08 | -0.0081 | 0.0054 | 0.134 | 31.6306092 |
| PE | MDD | rs117210485 | 9 | A | G | 0.115123 | 0.166552 | 0.0242352 | 6.31684E-12 | 0.0013 | 0.0161 | 0.9362 | 47.2283223 |
| PE | MDD | rs117468318 | 12 | T | A | 0.0163128 | 0.421067 | 0.0570362 | 1.55418E-13 | -0.0055 | 0.019 | 0.773499 | 54.5000971 |
| PE | MDD | rs143620474 | 9 | A | G | 0.0183405 | 0.305466 | 0.0539929 | 1.53564E-08 | 0.0505 | 0.0213 | 0.01798 | 32.0072585 |
| PE | MDD | rs2066865 | 4 | A | G | 0.301831 | 0.243169 | 0.01676 | 1.06439E-47 | 0.0018 | 0.0051 | 0.7166 | 210.505971 |
| PE | MDD | rs28584824 | 4 | A | C | 0.0855593 | -0.166105 | 0.0297625 | 2.39095E-08 | -0.0161 | 0.0125 | 0.1976 | 31.1474591 |
| PE | MDD | rs3756011 | 4 | A | C | 0.42911 | 0.23942 | 0.0158487 | 1.46622E-51 | 0.0046 | 0.0044 | 0.2989 | 228.207332 |
| PE | MDD | rs6025 | 1 | T | C | 0.0199477 | 0.522198 | 0.049121 | 2.14141E-26 | 0.0048 | 0.0139 | 0.7326 | 113.013946 |
| PE | MDD | rs62350309 | 4 | G | A | 0.102397 | -0.191477 | 0.0275962 | 3.96187E-12 | 0.0065 | 0.01 | 0.5134 | 48.1427275 |
| PE | MDD | rs78807356 | 11 | T | G | 0.00653341 | 0.502582 | 0.0854854 | 4.12373E-09 | 0.0267 | 0.0135 | 0.0482203 | 34.564159 |
| PE | MDD | rs80137017 | 10 | T | C | 0.107694 | -0.216462 | 0.026977 | 1.02376E-15 | 0 | 0.0065 | 0.9997 | 64.3831261 |
| DVT | MDD | rs13377102 | 10 | A | T | 0.109833 | -0.233421 | 0.0270701 | 6.53281E-18 | -0.0012 | 0.0063 | 0.845 | 74.3525156 |
| DVT | MDD | rs2066865 | 4 | A | G | 0.30142 | 0.18836 | 0.0170559 | 2.3518E-28 | 0.0018 | 0.0051 | 0.7166 | 121.961742 |
| DVT | MDD | rs2289252 | 4 | T | C | 0.424087 | 0.203856 | 0.0160345 | 4.96592E-37 | 0.0047 | 0.0044 | 0.2868 | 161.633616 |
| DVT | MDD | rs2420135 | 1 | G | A | 0.0858306 | 0.164265 | 0.0275011 | 2.32879E-09 | -0.0053 | 0.0093 | 0.5686 | 35.6767662 |
| DVT | MDD | rs4135105 | 12 | T | C | 0.0158018 | 0.477255 | 0.0567309 | 4.00959E-17 | -0.0224 | 0.0219 | 0.3067 | 70.7712985 |
| DVT | MDD | rs576123 | 9 | T | C | 0.56524 | -0.302516 | 0.015865 | 4.64408E-81 | 0.0077 | 0.0046 | 0.0953191 | 363.590128 |
| DVT | MDD | rs6025 | 1 | T | C | 0.0197631 | 1.09142 | 0.0419097 | 1.65577E-149 | 0.0048 | 0.0139 | 0.7326 | 678.18823 |
| DVT | MDD | rs62350309 | 4 | G | A | 0.102405 | -0.159746 | 0.027555 | 6.73705E-09 | 0.0065 | 0.01 | 0.5134 | 33.6089303 |
| DVT | MDD | rs7135039 | 12 | T | C | 0.35221 | 0.0938645 | 0.0168191 | 2.39376E-08 | -0.0068 | 0.0045 | 0.1282 | 31.1453183 |
| DVT | MDD | rs78807356 | 11 | T | G | 0.00645538 | 0.633897 | 0.083413 | 2.97303E-14 | 0.0267 | 0.0135 | 0.0482203 | 57.7517897 |
| DVT | MDD | rs9863058 | 3 | T | C | 0.367777 | -0.100174 | 0.0167562 | 2.25455E-09 | 0.0025 | 0.0048 | 0.5926 | 35.7399958 |

MDD, major depressive disorder; VTE, venous thromboembolism; PE, pulmonary embolism; DVT, deep venous thrombosis.

**Supplementary Table 5.** The results of MR-PRESSO and the MR statistical power

| Exposure | Outcome | MR-PRESSO global test (Outliers from MR-PRESSO) | RSSobs | Global Test`$Pvalue | OR (80% power) |
| --- | --- | --- | --- | --- | --- |
| MDD | VTE | NA | 56.201 | 0.053 | ≥ 1.365 or ≤ 0.645 |
| MDD | PE | NA | 36.769 | 0.6222 | ≥ 1.520 or ≤ 0.490 |
| MDD | DVT | NA | 56.201 | 0.051 | ≥ 1.526 or ≤0.490 |
| VTE | MDD | NA | 18.079 | 0.5286 | ≥ 1.076 or ≤ 0.928 |
| PE | MDD | NA | 10.673 | 0.4684 | ≥ 1.123 or ≤ 0.886 |
| DVT | MDD | NA | 16.022 | 0.252 | ≥ 1.090 or ≤ 0.915 |

MDD, major depressive disorder; VTE, venous thromboembolism; PE, pulmonary embolism; DVT, deep venous thrombosis; RSSobs, observed residual sum of squares; OR, odds ratio.

**Supplementary Table 6.** Associations of genetically determined MDD with VTE

| Exposure | Outcome | Mendelian Randomization Method | No.of SNPs used | OR | 95%CI | P value | Cochran's Q (p value) | MR-Egger intercept (P value) |
| --- | --- | --- | --- | --- | --- | --- | --- | --- |
| MDD | VTE | IVW | 37 | 0.936 | (0,736, 1.190) | 0.59 | 51.755(0.043) | -0.023(0.336) |
|  |  | Weighted median | 37 | 1.075 | (0.794,1.455) | 0.639 |  |  |
|  |  | Simple mode | 37 | 1.3 | (0.626, 2.700) | 0.486 |  |  |
|  |  | Weighted mode | 37 | 1.275 | (0.635, 2.559) | 0.498 |  |  |
|  |  | MR-Egger | 37 | 2.097 | (0.410, 10.537) | 0.383 |  |  |
| MDD | PE | IVW | 37 | 1.31 | (1.073, 1.598) | 0.0077 | 51.755(0.043) | -0.00408 (0.841) |
|  |  | Weighted median | 37 | 1.233 | (0.931, 1.632) | 0.143 |  |  |
|  |  | Simple mode | 37 | 1.039 | (0.571, 1.889) | 0.902 |  |  |
|  |  | Weighted mode | 37 | 1.131 | (0.639, 2.001) | 0.676 |  |  |
|  |  | MR-Egger | 37 | 1.503 | (0.392, 5.757) | 0.556 |  |  |
| MDD | DVT | IVW | 37 | 0.936 | (0.736, 1.190) | 0.59 | 51.755(0.043) | -0.023 (0.336) |
|  |  | Weighted median | 37 | 1.075 | (0.789, 1.465) | 0.647 |  |  |
|  |  | Simple mode | 37 | 1.3 | (0.602, 2.806) | 0.508 |  |  |
|  |  | Weighted mode | 37 | 1.275 | (0.658, 2.471) | 0.476 |  |  |
|  |  | MR-Egger | 37 | 2.079 | (0.410, 10.537) | 0.382 |  |  |

MDD, major depressive disorder; VTE, venous thromboembolism; PE, pulmonary embolism; DVT, deep vein thrombosis; IVW, inverse-variance weighted; OR, odds ratio; CI, confidence interval.

**Supplementary Table 7.** Associations of genetically determined VTE with MDD

| Exposure | Outcome | Mendelian Randomization Method | No.of SNPs used | OR | 95%CI | P value | Cochran's Q (p value) | MR-Egger intercept (P value) |
| --- | --- | --- | --- | --- | --- | --- | --- | --- |
| VTE | MDD | IVW | 17 | 1.004 | (0.989, 1.020) | 0.597 | 15.197 (0.51) | -0.000124 (0.965) |
|  |  | Weighted median | 17 | 1.006 | (0.984, 1.028) | 0.587 |  |  |
|  |  | Simple mode | 17 | 0.995 | (0.960, 1.032) | 0.789 |  |  |
|  |  | Weighted mode | 17 | 1.008 | (0.980, 1.036) | 0.595 |  |  |
|  |  | MR-Egger | 17 | 1.005 | (0.979, 1.031) | 0.736 |  |  |
| PE | MDD | IVW | 10 | 1.019 | (0.999, 1.039) | 0.066 | 9.475 (0.395) | -0.00577 (0.543) |
|  |  | Weighted median | 10 | 1.01 | (0.985, 1.035) | 0.453 |  |  |
|  |  | Simple mode | 10 | 1.007 | (0.975, 1.040) | 0.699 |  |  |
|  |  | Weighted mode | 10 | 1.011 | (0.985, 1.037) | 0.442 |  |  |
|  |  | MR-Egger | 10 | 1.04 | (0.972, 1.112) | 0.285 |  |  |
| DVT | MDD | IVW | 11 | 0.9997 | (0.984, 1.016) | 0.971 | 12.481 (0.254) | -0.0044 (0.223) |
|  |  | Weighted median | 11 | 1.004 | (0.985, 1.025) | 0.657 |  |  |
|  |  | Simple mode | 11 | 0.972 | (0.940, 1.005) | 0.121 |  |  |
|  |  | Weighted mode | 11 | 1.004 | (0.980, 1.029) | 0.741 |  |  |
|  |  | MR-Egger | 11 | 1.014 | (0.990, 1.040) | 0.329 |  |  |

MDD, major depressive disorder; VTE, venous thromboembolism; PE, pulmonary embolism; DVT, deep vein thrombosis; IVW, inverse-variance weighted; OR, odds ratio; CI, confidence interval.


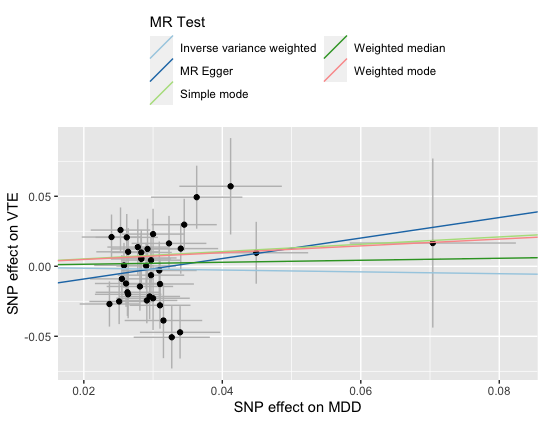


**Supplementary Figure 1.** Scatterplots comparing the strength of the SNP-exposure (MDD) and SNP-VTE associations. The lines indicate the estimated effect sizes by four Mendelian randomization methods (inverse‐variance weighted (IVW), Simple mode, MR Egger, Weighted median, and Weighted mode)


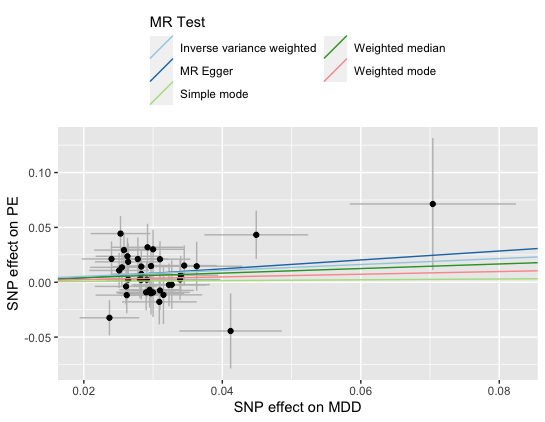


**Supplementary Figure 2.** Scatterplots comparing the strength of the SNP-exposure (MDD) and SNP-PE associations. The lines indicate the estimated effect sizes by four Mendelian randomization methods (inverse‐variance weighted (IVW), Simple mode, MR Egger, Weighted median, and Weighted mode)


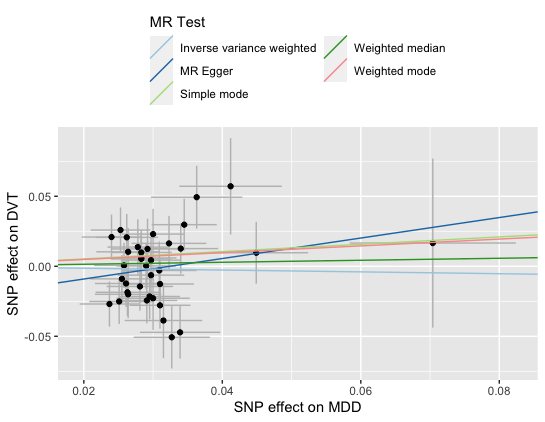


**Supplementary Figure 3.** Scatterplots comparing the strength of the SNP-exposure (MDD) and SNP-DVT associations. The lines indicate the estimated effect sizes by four Mendelian randomization methods (inverse‐variance weighted (IVW), Simple mode, MR Egger, Weighted median, and Weighted mode)


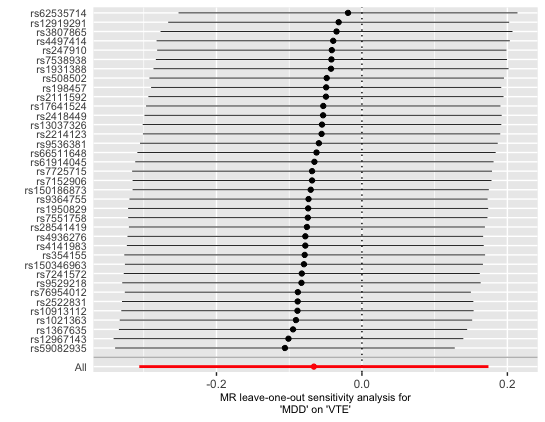


**Supplementary Figure 4.** Leave-one-out meta-analysis. The leave-one-out plot visualized how the causal estimates (point with horizontal line) for the effect of MDD on VTE were influenced by the removal of single variant

**
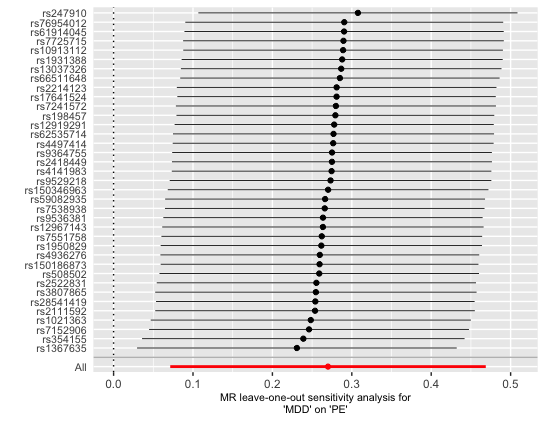
**

**Supplementary Figure 5.** Leave-one-out meta-analysis. The leave-one-out plot visualized how the causal estimates (point with horizontal line) for the effect of MDD on PE were influenced by the removal of single variant


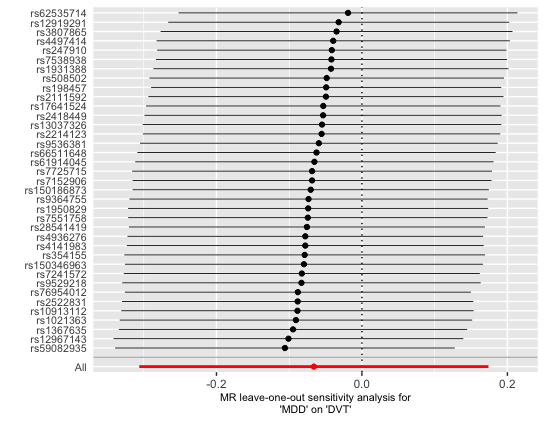


**Supplementary Figure 6**. Leave-one-out meta-analysis. The leave-one-out plot visualized how the causal estimates (point with horizontal line) for the effect of MDD on DVT were influenced by the removal of single variant


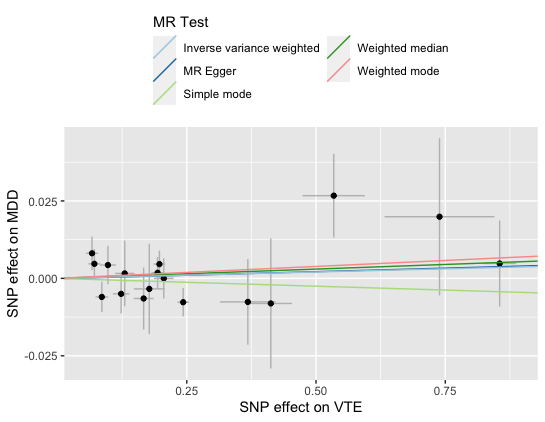


**Supplementary Figure 7.** Scatterplots comparing the strength of the SNP-exposure (VTE) and SNP-MDD associations. The lines indicate the estimated effect sizes by four Mendelian randomization methods (inverse‐variance weighted (IVW), Simple mode, MR Egger, Weighted median, and Weighted mode)


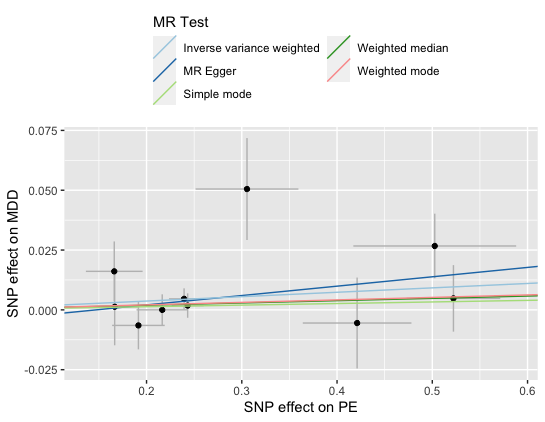


**Supplementary Figure 8.** Scatterplots comparing the strength of the SNP-exposure (PE) and SNP-MDD associations. The lines indicate the estimated effect sizes by four Mendelian randomization methods (inverse‐variance weighted (IVW), Simple mode, MR Egger, Weighted median, and Weighted mode)


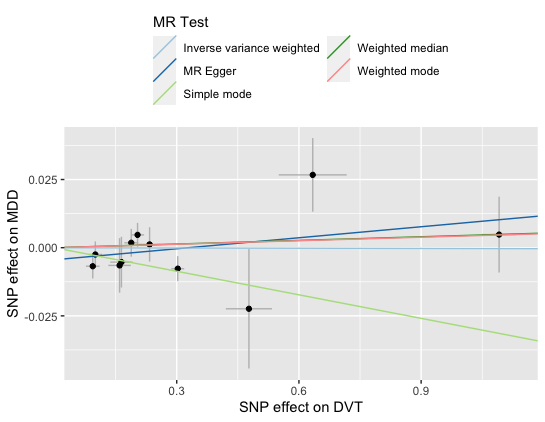


**Supplementary Figure 9.** Scatterplots comparing the strength of the SNP-exposure (PE) and SNP-MDD associations. The lines indicate the estimated effect sizes by four Mendelian randomization methods (inverse‐variance weighted (IVW), Simple mode, MR Egger, Weighted median, and Weighted mode)

**
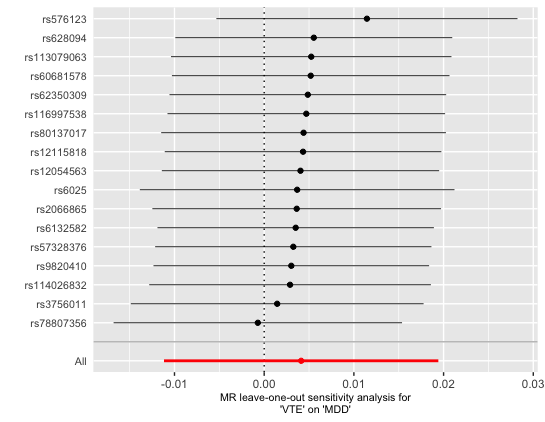
**

**Supplementary Figure 10.** Leave-one-out meta-analysis. The leave-one-out plot visualized how the causal estimates (point with horizontal line) for the effect of VTE on MDD were influenced by the removal of single variant

**
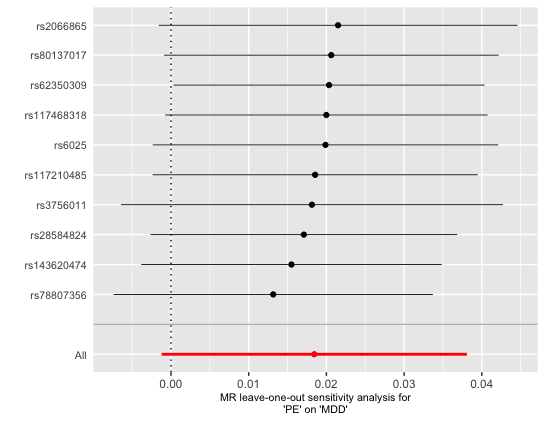
**

**Supplementary Figure 11.** Leave-one-out meta-analysis. The leave-one-out plot visualized how the causal estimates (point with horizontal line) for the effect of PE on MDD were influenced by the removal of single variant


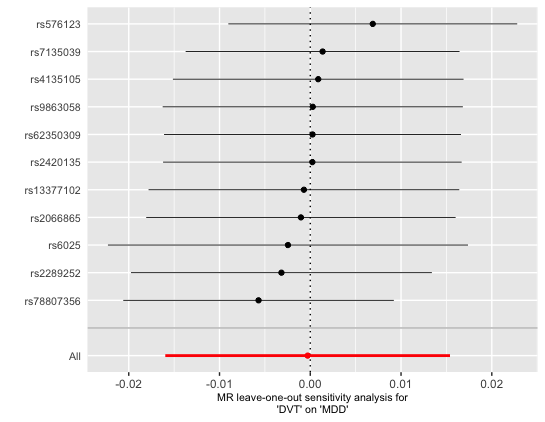


**Supplementary Figure 12**. Leave-one-out meta-analysis. The leave-one-out plot visualized how the causal estimates (point with horizontal line) for the effect of DVT on MDD were influenced by the removal of single variant
