# Supplementary figures and images for: Causal association between major depressive disorder and venous thromboembolism: a bidirectional mendelian randomization study
Source: Front Genet. 2024 Jun 25;15:1383333. doi: 10.3389/fgene.2024.1383333 (PMC11231919; doi:10.3389/fgene.2024.1383333)

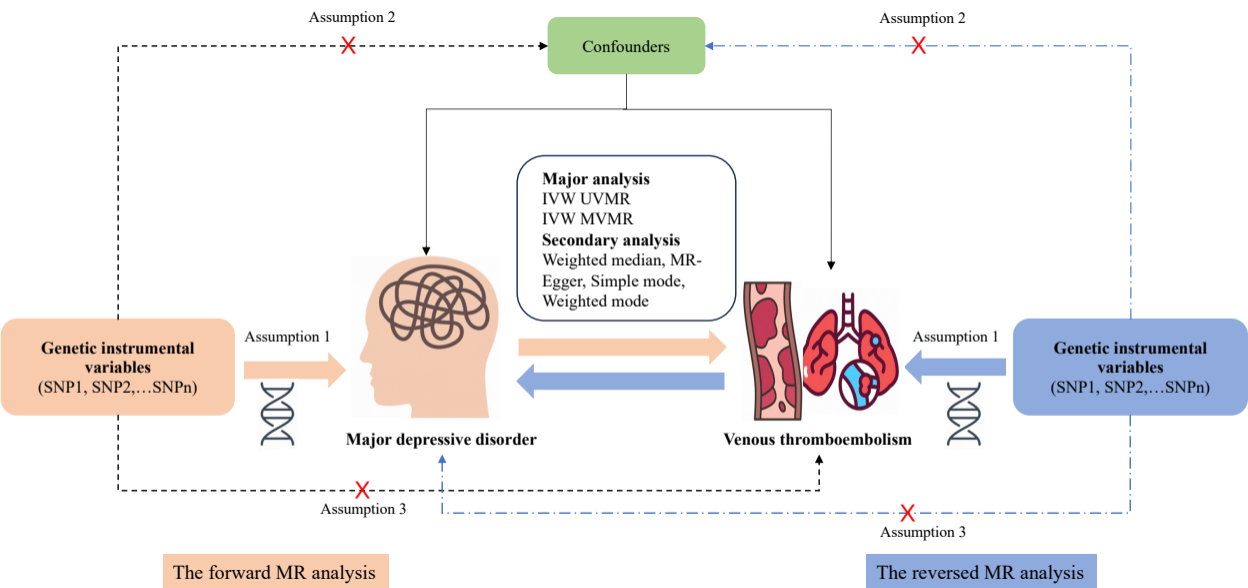

Supplement: Supplementary file 2 [file Image1.pdf]
